# The NMDA receptor regulates integrin activation, ATP release and arterial thrombosis through store-operated Ca^2+^ entry in platelets

**DOI:** 10.3389/fcvm.2023.1171831

**Published:** 2023-05-12

**Authors:** Friedrich Reusswig, Münteha Yilmaz, Marius Brechtenkamp, Irena Krueger, Lisa Maria Metz, Nikolaj Klöcker, Eckhard Lammert, Margitta Elvers

**Affiliations:** ^1^ Department of Vascular- and Endovascular Surgery, University Hospital Düsseldorf, Heinrich-Heine University, Düsseldorf, Germany; ^2^ Institute of Neural and Sensory Physiology, Medical Faculty and University Hospital Düsseldorf, Heinrich-Heine University, Düsseldorf, Germany; ^3^ Institute for Vascular and Islet Cell Biology, German Diabetes Center (DDZ), Leibniz Center for Diabetes Research at Heinrich-Heine University, Düsseldorf, Germany; ^4^ German Center for Diabetes Research (DZD e.V.), Helmholtz Zentrum München, Neuherberg, Germany; ^5^ Institute of Metabolic Physiology, Heinrich Heine University, Düsseldorf, Germany

**Keywords:** platelets, NMDAR, integrin, ATP, thrombosis, calcium

## Abstract

**Introduction:**

Platelet activation and thrombus formation is crucial for hemostasis, but also trigger arterial thrombosis. Calcium mobilization plays an important role in platelet activation, because many cellular processes depend on the level of intracellular Ca^2+^ ([Ca^2+^](i)), such as integrin activation, degranulation, cytoskeletal reorganization. Different modulators of Ca^2+^ signaling have been implied, such as STIM1, Orai1, CyPA, SGK1, etc. Also, the N-methyl-D-aspartate receptor (NMDAR) was identified to contribute to Ca^2+^ signaling in platelets. However, the role of the NMDAR in thrombus formation is not well defined.

**Methods:**

*In vitro* and *in vivo* analysis of platelet-specific NMDAR knock-out mice.

**Results:**

In this study, we analyzed *Grin1*^*fl/fl*^*-Pf4-Cre*^*+*^ mice with a platelet-specific knock-out of the essential GluN1 subunit of the NMDAR. We found reduced store-operated Ca^2+^ entry (SOCE), but unaltered store release in GluN1-deficient platelets. Defective SOCE resulted in reduced Src and PKC substrate phosphorylation following stimulation of glycoprotein (GP)VI or the thrombin receptor PAR4 followed by decreased integrin activation but unaltered degranulation. Consequently, thrombus formation on collagen under flow conditions was reduced *ex vivo*, and *Grin1*^*fl/fl*^*-Pf4-Cre*^*+*^ mice were protected against arterial thrombosis. Results from human platelets treated with the NMDAR antagonist MK-801 revealed a crucial role of the NMDAR in integrin activation and Ca^2+^ homeostasis in human platelets as well.

**Conclusion:**

NMDAR signaling is important for SOCE in platelets and contributes to platelet activation and arterial thrombosis. Thus, the NMDAR represents a novel target for anti-platelet therapy in cardiovascular disease (CVD).

## 1. Introduction

Platelets are anuclear cells with a crucial role in hemostasis and thrombosis. Platelet activation and aggregation is important to stop bleeding following vessel injury, but can also trigger pathological thrombus formation to provoke thrombosis. Consequently, platelets play a dominant role in cardiovascular diseases including myocardial infarction, stroke or abdominal aortic aneurysm (1–6). Cytosolic calcium is a second messenger important for many (patho-) physiological processes in platelets (7), including integrin activation, cytoskeletal reorganization, degranulation, pro-coagulant activity and thrombus formation (8–11). Ca^2+^ signaling is activated through activation of GPVI, thrombin receptors and purinergic receptors including P2Y1, P2Y12 and P2X1 at the platelet membrane leading to intracellular signaling cascades including the phosphorylation of PLC (8, 12). A variety of hormones and growth factors stimulate the hydrolysis of phosphatidylinositol 4, 5-bisphosphate (PIP2) by PLC, thereby producing two different messengers: Diacylglycerol (DAG) and inositol 1,4,5-trisphosphate (IP3) (13). IP3 binds and activates its receptor (IP3R), which is localized in the dense tubular system (DTS) membrane (14). Subsequently, Ca^2+^ is released from the DTS, resulting in a transient depletion of intraluminal Ca^2+^. Decreased Ca^2+^ levels activate stromal interaction molecule 1 (STIM1) at the DTS membrane. This induces STIM1 oligomer aggregation and its translocation to the plasma membrane (15). At this site, STIM1 binds to ORAI1 resulting in its activation. The activation of ORAI1 induces shape changes, which allows Ca^2+^ to pass into the cytosol, which stimulates the process termed as store-operated-Ca^2+^-entry (SOCE) and increases the intracellular Ca^2+^ level ([Ca2+](i)) in platelets. The increase activates multiple Ca^2+^-dependent targets. The increased Ca^2+^ level in the cell is also used as a Ca^2+^ source to replenish the intraluminal Ca^2+^ level in the DTS, for which the sarco (endo) plasmic reticulum calcium-ATPase 2 (SERCA2) is responsible (11). Transient receptor potential (TRP) proteins form a superfamily of cation channels that are expressed in platelets and many other cell types. Beside ORAI1 and STIM1, TRP channels regulate Ca^2+^ entry and [Ca^2+^](i) dynamics through different mechanisms including membrane depolarization or store- and receptor-operated mechanisms (16).

In recent years, N-methyl-D-aspartate receptors (NMDARs) were identified to contribute to Ca^2+^ signaling in platelets. NMDARs belong to the family of ionotropic glutamate receptors (iGluRs). They assemble from tetrameric combinations of GluN1, GluN2, and GluN3 subunits, with GluN1 being obligatory for channel function. Distinct from other iGluR subfamilies, most NMDARs show relevant Ca^2+^ permeability and exhibit a voltage-dependent block by extracellular Mg^2+^ ions. Activation of most NMDAR subunit combinations requires binding of the agonist glutamate to GluN2 and the co-agonists glycine or D-serine to GluN1 subunits. An exception form complexes from GluN1 and GluN3, lacking a glutamate-binding site, which serve as glycine-activated cation channels (17–20). NMDARs are particularly known for their role in the brain and serve as signaling pathway for glutamate-mediated intercellular communication. However, NMDARs are also active in non-neuronal cells (21) playing an important role in proliferation, cell growth and differentiation (22), actin rearrangement (23) cell adhesion and migration (24) as well as in regulation of hormone secretion (25). Moreover, the NMDAR is expressed in the heart and in the vasculature and NMDAR signaling might contribute to acute cardiovascular effects of ethanol (26). NMDARs are also involved in platelet function and pro-platelet formation (27, 28). However, the precise role of NMDARs in platelets and the contribution of NMDAR signaling to thrombosis is not well-defined to date. Therefore, we have analyzed platelet-specific NMDAR deficient mice (*Grin1^fl/fl^-Pf4-Cre^+^*) to identify NMDAR mediated activation and Ca^2+^ signaling in platelets important for hemostasis and thrombosis *in vitro* and *in vivo*.

## 2. Materials and methods

### 2.1. Chemicals and antibodies

For platelet isolation apyrase (Grade II from potato, #A7646, Sigma-Aldrich) and prostacyclin (#P5515, Calbiochem,) were used. Platelets were activated with collagen-related peptide (CRP; Richard Farndale, University of Cambridge, Cambridge, UK), adenosine diphosphate (ADP; #A2754, Sigma-Aldrich), the thromboxane A2 analogue U46619 (U46; #1932, Tocris), PAR4 peptide (PAR4; St. Louis, Missouri, MO, USA), thrombin (Thr; #10602400001, Roche Diagnostics, Germany), lipopolysaccharide (LPS, #L4524, Sigma-Aldrich) or convulxin (CVX, ALX-350-100-C050, ENZO Life Sciences). For immunoblotting antibodies targeting total Src (#2109, Cell Signaling, 1:1,000) and phosphorylated Src (#59548, Cell Signaling, 1:1,000), total Syk (#2712, Cell Signaling, 1:1,000), and phosphorylated Syk (#2710, Cell Signaling, 1:1,000), total Pannexin-1 (Panx1, #91137S, Cell Signaling, 1:1,000) and phosphorylated Panx1 (pPanx1 Tyr^198^, #ABN1681, Merck, 1:1,000), total Akt (Cell Signaling, #9272S) and phosphorylated Akt (pAkt Ser473, Cell Signaling, #9271S), β-Actin (Cell Signaling, #4967), glyceraldehyde 3-phosphate dehydrogenase (GAPDH, #2118S, Cell Signaling, 1:1,000), phospho-PKC substrate motif [(R/K)XpSX(R/K)] (Cell Signaling, #6967), and horseradish peroxidase (HRP)-linked anti-rabbit secondary antibodies (#7074S, Cell Signaling, 1:2,500) were used. Additionally and anti GLUN1-Antibody (#5704, Cell Signaling; 1:500) was used for detection of Grin1 expression in platelets. For flow cytometric analysis fluorophore conjugated antibodies labelling P-selectin (murine: CD62P, Wug.E9-FITC, #D200, Emfret Analytics; human: PE mouse anti-human P-Selectin, BD Biosciences), active integrin αIIbβ3 (murine: JON/A-PE, #D200; Emfret Analytics; human: FITC mouse anti-human PAC-1; BD Biosciences), GPIbα (CD42b, Xia.G5-PE, #M040-2 Emfret Analytics), GPVI (JAQ1-FITC, #M011-1, Emfret Analytics), integrin α5 (CD49e, Tap.A12-FITC, # M080-1, Emfret Analytics) and integrin β3 (CD61, Luc.H11-FITC, #M031-1, Emfret Analytics) were used. For inhibition experiments with human platelets, the inhibitor MK-801 (M107; Sigma-Aldrich) was used in indicated concentrations.

### 2.2. Human blood samples and ethic votes

Fresh citrate-anticoagulated blood (BD-Vacutainer®; Becton, Dickinson and Company; #367714) was collected from healthy volunteers. All healthy volunteers provided their written informed consent to participate in this study according to the Ethics Committee of the University Clinic of Duesseldorf, Germany (2018-140-kFogU, study number: 2018064710). This study was conducted according to the Declaration of Helsinki and the International Council for Harmonization Guidelines on Good Clinical Practice.

### 2.3. Human platelet isolation

For human platelet isolation, citrate-anticoagulated whole blood was centrifuged at 200×g for 10 min at RT and slowly decelerated. Subsequently, the upper phase, platelet-rich plasma (PRP) was collected and transferred into 1 ml PBS (pH 6.5). Platelet activation was inhibited by the addition of 2.5 U/ml apyrase and 1 µM prostaglandin E1 (PGE1). After centrifugation at 1,000×g for 6 min the supernatant was discarded and the platelet pellet was dissolved in human Tyrode's buffer (pH 7.4; 140 mM NaCl_2_, 2.8 mM KCl, 12 mM NaHCO_3_, 0.5 mM Na_2_HPO_4_, 5.5 mM glucose). For cell count determination, a 1:10 dilution in Tyrode's buffer was measured using a hematology analyzer (Sysmex—KX21N; Nordstedt, Germany). Cell count was set according to the experimental design by diluting with human Tyrode's buffer. MK-801 was preincubated for 15 min at 37°C for determination of altered platelet activation.

### 2.4. Animals

Pathogen-free *Grin1^fl/fl^* (B6.129S4-Grin1tmStl/J) mice were provided by Dr. Eckhardt Lammert and cross-bread with PF4-Cre mice that were purchased from the Jackson Laboratory [C57BL/6-Tg (Pf4-cre) Q3Rsko/J], to generate megakaryocyte/platelet specific deletion of Grin1. The *Grin1^fl/fl^-Pf4-Cre^+^* and *Grin1^fl/fl^-Pf4-Cre^−^* mice were already described and characterized by Hearn and colleagues (28).

*In vivo* thrombus formation assays and tail bleeding time were performed only in male mice aged 10–12 weeks (3–4 weeks for model of mesenteric arteries). All other experiments were conducted with male and female mice with an age of 2–4 months. Mice were maintained in an environmentally controlled room at 22 ± 1°C with a 12 h day-night cycle. Mice were housed in Macrolon cages type III with *ad libitum* access to food (standard chow diet) and water. All animal procedures were conducted according to the Declaration of Helsinki and are conform to the guidelines from Directive 2010/63/EU of the European Parliament on the protection of animals used for scientific purposes. All animal experiments were approved by the Ethics Committee of the State Ministry of Agriculture, Nutrition and Forestry State of North Rhine-Westphalia, Germany (Reference number: AZ 81-02.05.40.21.041; AZ 81-02.04.2021.A329).

### 2.5. Tail bleeding time

10 to 12-week-old mice were anesthetized by a singular intraperitoneal (i.p.) injection of Ketamine (100 mg/kg body weight, Ketaset®, company: Zoetis, Malakoff, France) and Xylazine (10 mg/kg body weight, Xylazin, company: WDT, Ulft, Netherlands). After progressing successfully anesthesia, the tail tip was cut with a scalpel at a position where the diameter of the tail is 2.25 – 2.5 mm using a gauge. The tail was immersed in normal saline (37°C). The time from the incision to the cessation of bleeding was recorded (no blood flow for 1 min) as described previously (30). Euthanasia was performed by cervical dislocation.

### 2.6. Mouse model of arterial injury of mesenteric arterioles

Mice (3–4 weeks of age) were anesthetized by a singular intraperitoneal (i.p.) injection of Ketamine (100 mg/kg body weight, Ketaset®, company: Zoetis, Malakoff, France) and Xylazine (10 mg/kg body weight, Xylazin, company: WDT, Ulft, Netherlands). After progressing successfully anesthesia, a midline abdominal incision was done, the mesentery was exteriorized and arterioles free of fat tissue were injured by topical application of a filter paper saturated with 20% FeCl_3_ for 20 s. Thrombus formation was observed with a fluorescence microscope (40×, Zeiss, Axio Observer, Germany). Time until full occlusion of the vessel (when blood flow had stopped for more than 60 s) was measured. After 40 min without vessel occlusion, experiments were terminated. Euthanasia was performed by cervical dislocation.

### 2.7. Mouse model of arterial injury of the *A. carotis communis*

10 to 12-week-old mice were anesthetized by intraperitoneal administration of Ketamine and Xylazine as described above and the right A. carotis communis was prepared and placed into a flow probe (Transonic Systems, 0.5 PSB, AD Instruments). Before starting the measurement, the artery and the surrounding area were moisturized with 0.9% NaCl_2_ and blood flow was measured in ml/min. Then a small pad was placed under the artery below the measuring head and the environment of the artery was dried. A 0.5 × 1 mm filter paper (Whatmann No.1) saturated with 10% FeCl_3_ (Sigma-Aldrich) was placed at the area of the carotid artery below the measuring head for 3 min. After removing the filter paper, the environment of the artery was moisturized with 0.9% NaCl_2_ again. The formation of the thrombus and or occlusion of the artery was recorded by the software until 5 min after a firm occlusion (stopped blood flow) or if there is no occlusion until the measurement reached a duration of 30 min. Euthanasia was performed by cervical dislocation.

### 2.8. Murine blood collection and platelet isolation

Mice were placed for anesthesia with controlled supply of isoflurane (2%) and oxygen (1 L/min). The blood was collected by using glass capillaries covered with sodium heparin by puncturing the retrobulbar venous plexus. Murine platelet isolation was conducted as described elsewhere (29). Briefly, murine heparinized blood was centrifuged for 5 min at 250×g (at RT). The upper phase was collected into a new reaction tube and an additional centrifugation step at 50×g for 5 min at RT followed to prevent contamination of other blood cells. The PRP was collected in a fresh tube with the addition of apyrase (0.02 U/ml) and prostacyclin (0.5 µM; PGI2) to prevent platelet preactivation. PRP was washed two times at 650×g for 5 min with murine Tyrode's buffer [pH 7.35; 136 mM NaCl, 0.4 mM Na_2_HPO_4_, 2.7 mM KCl, 12 mM NaHCO_3_, 0.1% glucose, 0.35% bovine serum albumin (BSA)], containing apyrase and PGI2. Cell count was determined in a 1:10 dilution at using a hematology analyzer. For experimental use, the isolated platelets were resuspended in Tyrode's buffer supplemented with 1 mM CaCl_2_ (in absence of apyrase, PGI2 and BSA).

### 2.9. Immunoblotting

Murine platelets were isolated as described in 2.8. Isolated platelets (40 × 10^6^) were stimulated with CRP or thrombin for indicated time-points at 37°C for time dependent analysis. For concentration dependent analysis, CRP stimulation was terminated after 2 min, and PAR4 stimulation after 5 min. Platelet stimulation was terminated by adding 1× human lysis buffer (100 mM Tris-HCl, 725 mM NaCl_2_, 20 mM EDTA, 5% TritonX-100, complete protease inhibitor). Lysis was performed for 15 min at 4°C. Afterwards, 6× Laemmli buffer was added to the platelet lysates, followed by a denaturation step at 95°C for 5 min. The platelet lysates were stored at −70°C until use. For immunoblotting platelet lysates were separated by sodium dodecyl sulfate polyacrylamide gel electrophoresis (SDS-PAGE; 10% acrylamide). After gel separation using SDS-PAGE running buffer [pH 8.3; 25 mM Tris base, 192 mM glycine, and 0.1% (w/v) SDS] for 2.5 h (25 mA), the gels were blotted to a nitrocellulose membrane for 1 h (70 mA). Unspecific antibody binding was prevented by blocking the membranes in 5% non-fat dry milk in TBST (Tris-buffered saline containing 0.1% Tween20) for 1 h at RT. After 3× times washing n TBST the membrane was probed with specific antibody's listed under 2.1 at 4°C overnight. After washing, the membranes were probed with HRP-conjugated secondary antibody (#7074S, Cell Signaling, 1:2,500) for 1 h at RT. GAPDH (#2118S, Cell Signaling, 1:1,000) served as loading control. Bands were visualized by chemiluminescence detection reagents (BioRad, #1705061) and imaged using a FusionFX Chemiluminescence Imager System (Vilber). Quantification of immune-reactive band intensities was performed by using Image J sofware.

### 2.10. Determination of ATP release by enzyme-linked immunosorbent assay (ELISA)

Human and murine platelets were isolated and activated with CRP or thrombin as described above and fixed for 2 h at 4°C using 2% Paraformaldehyde. The platelets were centrifuged at 650×g and releasates were collected and stored at −70°C before use. ATP levels were quantified using the bioluminescence assay kit from Roche Diagnostics following the manufacturer's protocol.

### 2.11. Flow cytometry

Flow cytometry was performed as described elsewhere (30). Briefly, heparinized murine whole blood was washed three times with murine Tyrode's buffer by centrifugation at 650×g for 5 min. After washing the whole blood samples were diluted in murine Tyrode's buffer containing 1 mM CaCl_2_. Citrate-anticoagulated human blood samples were diluted in a 1:10 ratio with human Tyrode's buffer. For the analysis of platelet activation, samples were stimulated with indicated agonists and specifically labeled with antibodies against P-selectin and active integrin αIIbβ3 (JON/A) in a ratio of 1:10 for 15 min at 37°C. Reaction was stopped by adding 300 µl of PBS to all samples. To analyze glycoprotein (GP) expression, washed whole blood was labeled for GPIbα, GPVI, integrin α5, or integrin β3 in a ratio of 1:10 for 15 min at RT. In All samples the MFI (mean fluorescence intensity) of the platelet specific FSC/SSC population was analyzed using a FACSCalibur flow cytometer (BD Biosciences).

### 2.12. Determination of intracellular calcium mobilization

Murine and human platelets were isolated as described above and resuspended in respective Tyrode's buffer without Ca^2+^ and loaded with 5 µM Fura-2 acetoxymethyl ester (#65-0858-39; Invitrogen) and 0.2 µg/ml of Pluronic F-127 (P2443; Biotium) at 37°C for 20 min. Platelets were washed once and resuspended in Tyrode's buffer containing 0.5 mM EGTA (#3054; Roth) for determination of store release measurements. For detection of store operated calcium entry (SOCE) buffer containing 1 mM Ca^2+^ was used. After platelet activation with different agonists and co-incubation with MK-801 for human platelets, the Ca^2+^ response was measured under stirring conditions. A spectrofluorometer (FL 6500; PerkinElmer) at alternate excitation wavelengths of 340 and 380 nm was used. The ratio values of 340/380 nm were converted into Ca^2+^ concentrations (Ca^2+^) by lysis of platelets with Triton X-100 (T8787; Sigma-Aldrich) for detection of the maximum ratio value. Followed by adding of EGTA to determine a minimum ratio value. Measurements with thapsigargin (TG; T7458; Invitrogen) were performed in Ca^2+^-free Tyrode's buffer. Incubation of platelets with 5 µM TG was followed by the addition of 1 mM Ca^2+^ to measure Ca^2+^ influx in murine and human platelets.

### 2.13. Platelet aggregation and ATP release

Freshly isolated platelets from healthy volunteers were measured as percentage light transmission compared to human Tyrode's buffer (as = 100%) using Chrono-Log dual channel lumi-aggregometer (model 700) at 37°C stirring at 1,000 rpm. Agonists were used as indicated and human platelets were pre-incubated with MK-801 for 15 min at 37°C before measurement.

ATP release from human platelets was assessed applying a luciferin/luciferase bioluminescent assay and calculated using a provided ATP standard protocol (all Chrono-Log).

### 2.14. Thrombus formation under flow: flow chamber experiments

Cover slips (24 × 60 mm) were coated with 200 µg/ml fibrillary type I collagen (Nycomed) and blocked with 1% BSA. Subsequently, heparinized murine or citrate-anticoagulated human whole blood was filled in a 1 ml syringe and perfused over a collagen-coated surface at shear rate of 450, 700, 1,000 and 1,700 s^−1^ and platelet adhesion and aggregate formation was evaluated from five different microscopic areas (LD Plan-Neofluar x 40, Axio Observer D1; Carl Zeiss) using Image J Software.

### 2.15. Statistical analysis

Data are presented as arithmetic means ± SEM (Standard error of mean), statistical analysis was performed using GraphPad Prism 8 (version 8.4.3). Statistical differences were determined using a two-way or one-way ANOVA with a Sidak's multiple comparison post-hoc test, an unpaired multiple *t*-test or an unpaired student's *t*-test. Significant differences are indicated by asterisks (***P < 0.001; **P < 0.01; *P < 0.05).

## 3. Results

### 3.1. NMDAR in platelets contributes to store-operated Ca^2+^ entry (SOCE) but not to store release following platelet activation

The absence of the GluN1 subunit of the NMDAR on platelets isolated from *Grin1^fl/fl^-Pf4-Cre^+^* mice was confirmed (Supplementary Figure S1A). The number of red blood cells (RBCs) (Supplementary Figure S1B) and white blood cells (WBCs) (Supplementary Figure S1C) were unaltered in *Grin1^fl/fl^-Pf4-Cre^+^* mice compared to controls (*Grin1^fl/fl^-Pf4-Cre^−^* mice). In contrast to Hearn et al., we detected reduced platelet counts only by trend (Supplementary Figure S1D) while the mean platelet volume (Supplementary Figure S1E) was unaltered between groups. The exposure of different receptors at the platelet membrane including glycoproteins and integrins were unaltered in *Grin1^fl/fl^-Pf4-Cre^+^* mice compared to controls (*Grin1^fl/fl^-Pf4-Cre^−^* mice) (Supplementary Figure S1F).

**Figure 1:**
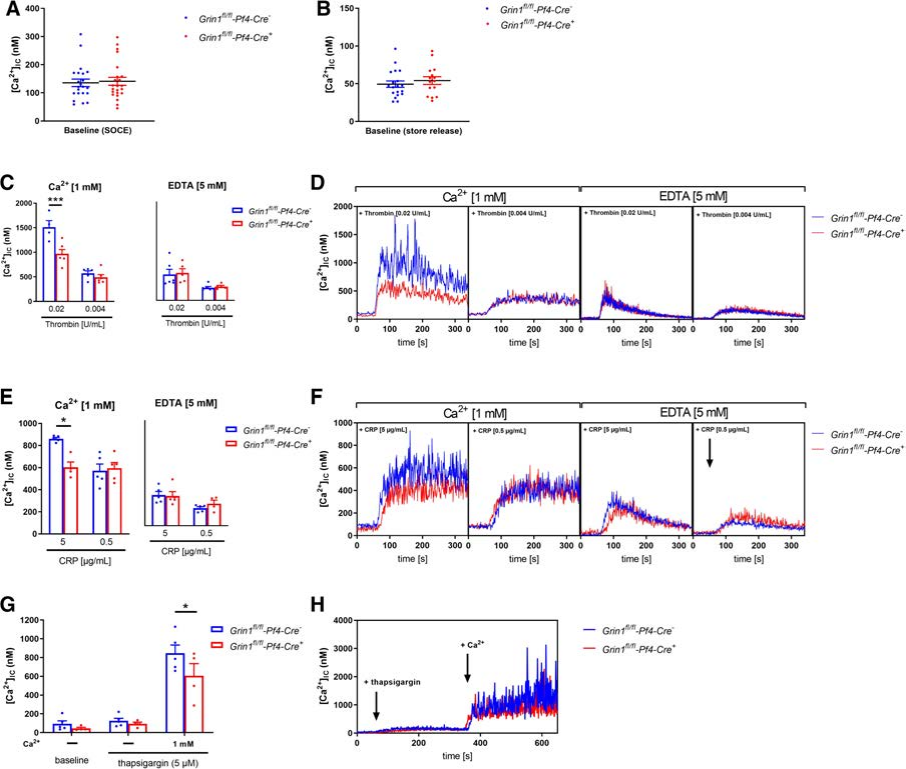
NMDAR regulates store operated calcium entry (SOCE) in murine platelets. (**A**) Determination of intracellular Ca^2+^ concentration [(Ca^2+^)_IC_] of platelets in buffer containing 1 mM Ca^2+^ for SOCE measurement (*n* = 22–23). (**B**) Determination of [Ca^2+^]_IC_ of platelets in buffer containing 5 mM EGTA for store-release measurement (*n* = 16–20). (**C**) Determination of SOCE and store-release in NMDAR deficient platelets after stimulation with thrombin compared to respective WT control platelets (*n* = 5–6). (**D**) Representative measurements of [Ca^2+^]_IC_ in the spectral fluorimeter after thrombin stimulation. (**E**) Determination of SOCE and store-release in *Grin1^fl/fl^-Pf4-Cre^+^*platelets after stimulation with collagen-related peptide (CRP) compared to respective WT control platelets (*n* = 5). (**F**) Representative measurements of [Ca^2+^]_IC_ in the spectral fluorimeter after CRP stimulation. (**G**) Determination of the [Ca^2+^]_IC_ using a sarcoplasmic/endoplasmic reticulum Ca^2+^-ATPases (SERCA-2) inhibitor thapsigargin following Ca^2+^ administration (*n* = 4–5). (**H**) Representative measurements of intracellular Ca^2+^ flux in the spectral fluorimeter after SERCA-2 inhibition via thapsigargin and Ca^2+^ exposure. Depicted are mean values + s.e.m.; *P < 0.05, ***P < 0.001 using two-way ANOVA followed by Sidak's multiple comparison test.

In a first step, we analyzed Ca^2+^ mobilization upon platelet activation using platelets from *Grin1^fl/fl^-Pf4-Cre^+^* and *Grin1^fl/fl^-Pf4-Cre^−^* mice because the NMDAR forms an ion channel pore that is selective for cations, in particular for Ca^2+^ and channel opening is induced by NMDAR activation. Baseline levels of [Ca^2+^](i) were unaltered in *Grin1^fl/fl^-Pf4-Cre^+^*compared to controls (*Grin1^fl/fl^-Pf4-Cre^−^)* (Figures 1A,B). Subsequent activation of platelets with thrombin resulted in intracellular Ca^2+^ mobilization in platelets. However, in the presence of extracellular Ca^2+^, the increase in intracellular Ca^2+^ [(Ca^2+^)i] was significantly reduced after stimulation of platelets with high doses of thrombin while no alterations have been detected in the absence of extracellular Ca^2+^ (i.e., in the presence of EDTA) (Figures 1C,D). Stimulation of platelets with CRP resulted in an increase in [Ca^2+^](i) in both, platelets from *Grin1^fl/fl^-Pf4-Cre^+^* and *Grin1^fl/fl^-Pf4-Cre^−^*mice. However, the increase of [Ca^2+^](i) was significantly reduced after stimulation of platelets with high doses of CRP in the presence of extracellular Ca^2+^ while no differences were detected in the absence of extracellular Ca^2+^ (i.e., in the presence of EDTA) (Figures 1E,F). Measurements of agonist-induced changes in [Ca^2+^]i revealed a clear reduction of store-operated Ca^2+^ entry (SOCE) but not of store release. These defects in extracellular Ca^2+^ mobilization in NMDAR deficient platelets suggested a general defect in Ca^2+^ homeostasis induced by NMDAR deficiency.

To test this hypothesis, we treated Fura-2–loaded platelets with the SERCA inhibitor thapsigargin (TG). Again, no differences in [Ca^2+^]i were detected at baseline (Figures 1G,H). In the absence of extracellular Ca^2+^, TG (5 µM) triggered a Ca^2+^ release from intracellular stores that was unaltered between platelets of both groups. However, the addition of extracellular Ca^2+^ induced SOCE that was significantly reduced in platelets from *Grin1^fl/fl^-Pf4-Cre^+^* compared to controls (*Grin1^fl/fl^-Pf4-Cre^−^)* (Figures 1G,H). Taken together, these results demonstrate that the NMDAR is involved in SOCE but not in store release, indicating a role for the NMDAR as a powerful Ca^2+^ regulator for extracellular Ca^2+^ mobilization in platelets.

### 3.2. NMDAR is important for integrin αIIbβ3 activation and ATP release of platelets

**Figure 2:**
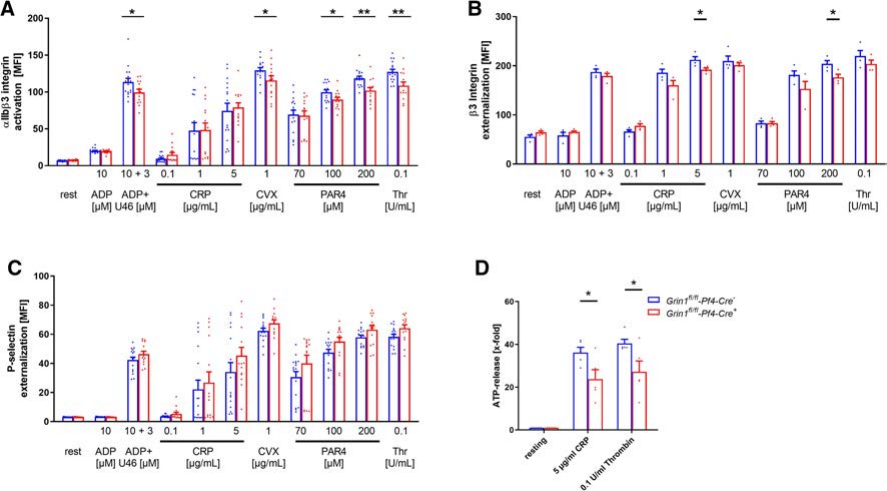
Loss of functional NMDAR leads to reduced αIIbβ3 integrin activation and ATP release of platelets. (**A**) Activation of αIIbβ3 integrin on the platelet surface with indicated agonists (*n* = 16). (**B**) Externalization upon platelet stimulation of the β3 Integrin subunit (*n* = 4). (**C**) Externalization of P-selectin on the platelet surface with indicated agonists (*n* = 16). (**D**) ATP-release measured in platelet releasates after activation with indicated agonists (*n* = 5). Depicted are mean values + s.e.m.; *P < 0.05, **P < 0.01 using unpaired students *t*-test. CVX, convulxin; CRP, collagen related peptide; U46, U46689 (Thromboxane A2 analog); Thr, thrombin; rest, resting state; PAR4, PAR4 activating peptide; ADP, adenosine-diphosphate.

Next, we analyzed platelet activation after stimulation of platelets with standard agonists. As shown in Figure 2A, the activation of integrin αIIbβ3 (fibrinogen receptor) was reduced after stimulation of platelets with ADP and U46619 (thromboxane analogue), the snake venom convulxin (CVX), PAR4 peptide and thrombin (Figure 2A). This might be due at least in part by reduced agonist-stimulated up-regulation of integrin αIIbβ3 at the platelet membrane (Figure 2B). In contrast, P-selectin exposure was not affected in GRIN1 deficient platelets compared to wildtype controls (*Grin1^fl/fl^-Pf4-Cre^−^)* (Figure 2C)*.* However, we detected reduced dense granule release as detected by levels of ATP following platelet activation (Figure 2D). These results suggest that the NMDAR in platelets is important for the activation of integrin αIIbβ3 and the release of ATP from dense granules.

### 3.3. Genetic deletion of NMDAR leads to reduced phosphorylation of Src and protein kinase (PK)C substrates in platelets

**Figure 3:**
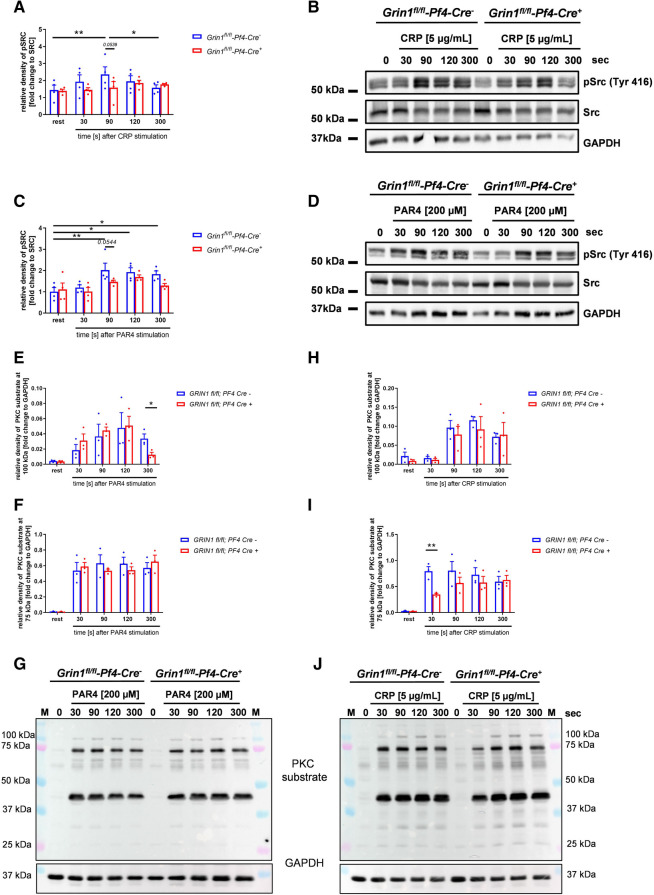
*Grin1^fl/fl^-Pf4-Cre^+^*platelets show attenuated Src and PKC substrate phosphorylation after stimulation. (**A**) Time dependent analysis of Src phosphorylation (Tyr416) after stimulation with collagen related peptide (CRP) of NMDAR deficient platelets (with (**B**) respective WT controls and representative blots (*n* = 4). (**C**) Time dependent analysis of Src phosphorylation (Tyr416) after stimulation with PAR4 activating peptide (PAR4) of NMDAR deficient platelets with (**D**) respective WT controls and representative blots (*n* = 5). (**E-G**) Time dependent analysis of PKC substrate phosphorylation after stimulation of platelets with collagen related peptide (CRP). Analysis of PKC substrate phosphorylation for (**E**) the 100 kDa signal, (**F**) the 75 kDa signal with (**G**) representative blots (*n* = 3). (**H–J**) Time dependent analysis of PKC substrate phosphorylation after stimulation of platelets with collagen related peptide (CRP). Analysis of PKC substrate phosphorylation for (**H**) the 100 kDa signal, (**I**) the 75 kDa signal with (**J**) representative blots (*n* = 3, GAPDH served as loading control). Depicted are mean values + s.e.m.; *P < 0.05, **P < 0.01 using two-way ANOVA followed by Sidak's multiple comparison test for time dependent comparison and unpaired students *t*-test for comparison at same stimulation time point.

Src family kinases (SFKs) are key regulators of NMDAR function and major points of convergence for neuronal transduction pathways (31, 32). To investigate if Src activation plays a role in NMDAR function in platelets as well, we performed Western blot analysis and analyzed the phosphorylation of Src at Tyr416. As shown in Figure 3A–D, we found delayed Src phosphorylation at Tyr416 in response to CRP or PAR4 peptide because full Src activation was detected after 90 s in control (*Grin1^fl/fl^-Pf4-Cre^−^)* and after 120 s in *Grin1^fl/fl^-Pf4-Cre^+^* platelets (Figures 3A–D). Furthermore, we analyzed PKC substrate serine phosphorylation after stimulation of platelets with PAR4 peptide and CRP, respectively. We detected reduced serine phosphorylation of PKC substrates with a size of 100 kDa following PAR4 peptide stimulation and with a size of 75 kDa following CRP stimulation of platelets (Figures 3E–J). However, no differences in serine phosphorylation of PKC substrates with a size of 40 kDa were found after platelet activation (Supplementary Figures S2A,B). Only resting platelets from Grin1 deficient mice showed reduced phosphorylation of PKC substrates with a size of 40 kDa (Supplementary Figure S2A). In the central nervous system, it is well known that one target of the NMDAR is pannexin-1 (Panx1). Therefore, we analyzed the phosphorylation of Panx1 as marker for Panx1 activation. Platelets were stimulated with CRP or PAR4 peptide using low and high doses of the agonist and analyzed by Western blot to determine the phosphorylation of Panx1 at Tyr198 known as marker for Panx1 activation (33). As shown in Supplementary Figure S2, no differences in Panx1 activation between platelets from *Grin1^fl/fl^-Pf4-Cre^+^* and *Grin1^fl/fl^-Pf4-Cre^−^* mice were observed following stimulation of platelets with CRP or PAR4 peptide (Supplementary Figures S2C,D). We next investigated the activation of Syk. As shown in Supplementary Figure S2, the phosphorylation of Syk was unaltered in platelets from *Grin1^fl/fl^-Pf4-Cre^+^* mice compared to controls (*Grin1^fl/fl^-Pf4-Cre^−^)* (Supplementary Figures S2E–H). Furthermore, the phosphorylation of Akt was unaltered as well as shown in Supplementary Figures S2I–L. Taken together, we provide first evidence that Src and different PKC substrates become phosphorylated/activated following NMDAR activation in platelets as already observed in the central nervous system.

### 3.4. Decreased Ca^2+^ mobilization and integrin activation results in reduced thrombus formation under flow conditions *ex vivo*

**Figure 4:**
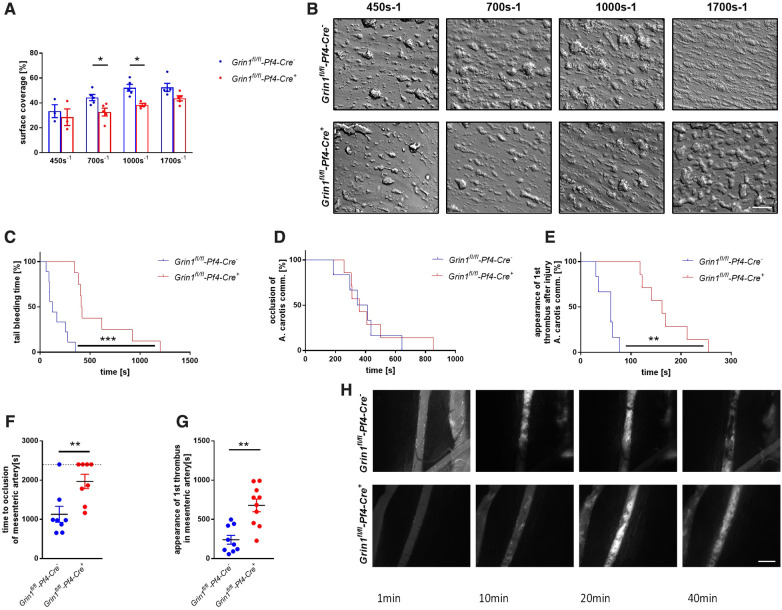
Genetic deletion of GRIN1 in platelets leads to reduced thrombus formation *in vivo* and prolonged bleeding times. (**A**) Analysis of thrombus formation at indicated shear rates on a collagen matrix with (**B**) representative images from platelet specific NMDAR deficient mice (*Grin1^fl/fl^-Pf4-Cre^+^*) and respective controls (*Grin1^fl/fl^-Pf4-Cre^−^)* (*n* = 3–6). (**C**) *In vivo* analysis of hemostatic dysfunction as determined by tail bleeding times (*n* = 9). (**D**) *In vivo* analysis of FeCl_3_-induced injury of the carotid artery via time to full occlusion of the artery and (**E**) analysis of beginning of thrombus formation (*n* = 6–10). (**F**) *In vivo* analysis of FeCl_3_-induced injury of the mesenteric artery via time to full occlusion of the artery and (**G**) analysis of beginning of thrombus formation (*n* = 8–9) with (**H**) representative microscopic images at indicated time points. *P < 0.05, **P < 0.01, ***P < 0.001 using unpaired student's *t*-Test (A, F and G). For analysis of Kaplan Meyer curves a log-rank test was used (C, D and E). Scale bars 50 µm (**B**) and 100 µm (**H**).

To analyze the consequences of NMDAR deficiency in platelets under more physiological conditions, we performed flow chamber experiments. To this end, we perfused whole blood of *Grin1^fl/fl^-Pf4-Cre^+^* and *Grin1^fl/fl^-Pf4-Cre^−^* mice over a collagen coated surface at different shear rates using a pulse-free pump. As shown in Figure 4, the deficiency of NMDAR in platelets resulted in reduced thrombus formation under flow conditions (Figures 4A,B). Significantly reduced surface coverage of three-dimensional thrombi was observed under a shear rate of 700 s^−1^ and 1,000 s^−1^. Reduced thrombus formation using a shear rate of 1,700 s^−1^ was only detected by trend (Figures 4A,B).

### 3.5. Platelet NMDAR is important for hemostasis and arterial thrombosis

To test, if defective extracellular Ca^2+^ mobilization and integrin activation in GluN1 deficient platelets affects thrombus formation *in vivo*, we performed a tail bleeding assay to analyze hemostasis in *Grin1^fl/fl^-Pf4-Cre^+^* mice. As expected, the time to stop bleeding after cutting the tail tip of mice was enhanced in *Grin1^fl/fl^-Pf4-Cre^+^* mice compared to controls (*Grin1^fl/fl^-Pf4-Cre^−^)* (Figure 4C). So far, no data exist about the role of platelet NMDAR in arterial thrombosis *in vivo*. Therefore, we analyzed *Grin1^fl/fl^-Pf4-Cre^+^* and *Grin1^fl/fl^-Pf4-Cre^−^* mice in two different mouse models of arterial thrombosis. In the first model, we induced the injury of the carotid artery by FeCl_3_. As shown in Figure 4D, the time to occlusion of the vessel was attenuated in *Grin1^fl/fl^-Pf4-Cre^+^* mice without reaching statistical significance. In contrast, an enhanced time period in *Grin1^fl/fl^-Pf4-Cre^+^* mice was detected when the appearance of first thrombi after vessel injury were analyzed (Figure 4E) suggesting attenuated thrombus formation after injury of the carotid artery in *Grin1^fl/fl^-Pf4-Cre^+^* mice. In a second arterial mouse model, injury of mesenteric arterioles was induced by topical application of FeCl_3_ (Figures 4F–H). Platelet-specific GluN1 deletion resulted in an enhanced occlusion time of the injured vessel. In addition, the appearance of first thrombi was attenuated. Taken together, these results suggest that platelet-specific deficiency of the GluN1 protects mice against arterial thrombosis but also prolongs bleeding times suggesting a delayed hemostasis in these mice.

### 3.6. NMDAR blocking in human platelets results in impaired SOCE

**Figure 5:**
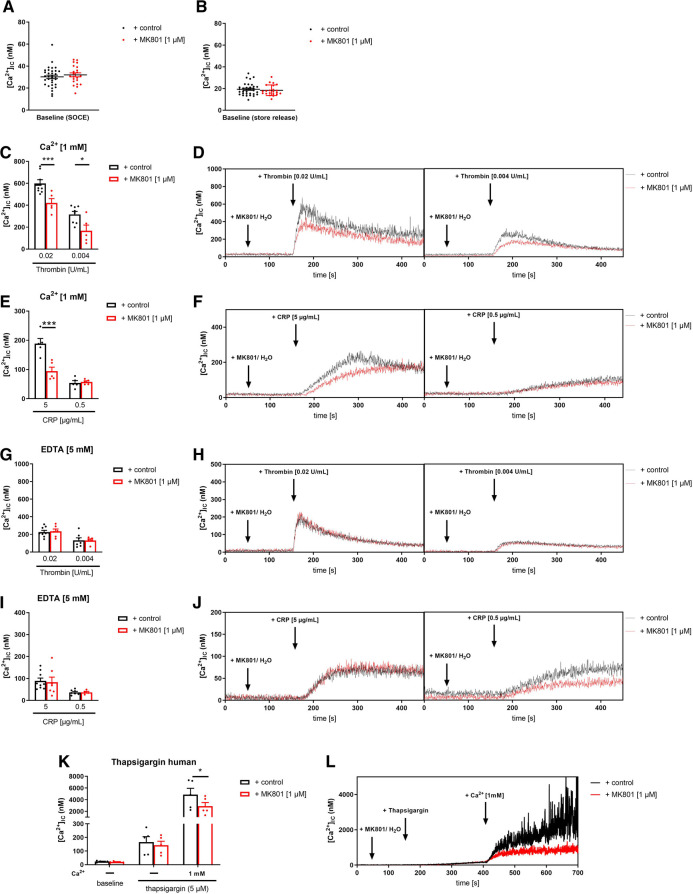
Inhibition of NMDAR leads to reduced store-operated-calcium-entry (SOCE) in human platelets. (**A**) Determination of intracellular Ca^2+^ concentrations [Ca^2+^](i) of platelets in buffer containing 1 mM Ca^2+^ for SOCE measurement after addition of MK-801 (*n* = 22–33). (**B**) Determination of [Ca^2+^](i) of platelets in buffer containing 5 mM EGTA for store-release measurement after addition of MK-801 (*n* = 22–33). (**C**) Determination of SOCE after NMDAR receptor blockade via MK-801 followed by thrombin stimulation (*n* = 5–8). (**D**) Representative measurements of [Ca^2+^](i) in the spectral fluorimeter after thrombin stimulation. (**E**) Determination of SOCE after MK-801 addition followed by stimulation with collagen-related peptide (CRP) compared to vehicle control treated platelets (*n* = 5). (**F**) Representative measurements of [Ca^2+^](i) in the spectral fluorimeter after CRP stimulation. (**G**) Determination of store release after NMDAR receptor blockade via MK-801 followed by thrombin stimulation (*n* = 5–8). (**H**) Representative measurements of [Ca^2+^](i) in the spectral fluorimeter after thrombin stimulation. (**I**) Determination of store release after MK-801 addition followed by stimulation with collagen-related peptide (CRP) compared to vehicle control treated platelets (*n* = 6–8). (**J**) Representative measurements of [Ca^2+^](i) in the spectral fluorimeter after CRP stimulation. (**K**) Determination of the [Ca^2+^](i) using the sarcoplasmic/endoplasmic reticulum Ca^2+^-ATPases (SERCA-2) inhibitor thapsigargin and the NMDAR receptor inhibitor MK-801 following Ca^2+^ administration (*n* = 5). (**L**) Representative measurements of intracellular Ca^2+^ flux in the spectral fluorimeter after SERCA-2 and NMDAR receptor inhibition via thapsigargin and MK-801. Depicted are mean values + s.e.m.; *P < 0.05, ***P < 0.001 using two-way ANOVA followed by Sidak's multiple comparison test.

In a first translational approach, we isolated platelets from healthy volunteers and blocked the NMDAR through the selective NMDA receptor antagonist MK-801. We incubated human platelets with different concentrations of MK-801 to test, if the inhibitor affects Ca^2+^ baseline levels in the presence or absence of extracellular Ca^2+^ (Figures 5A,B). No differences at baseline were observed, neither with 1, 10 or 100 µM MK-801 compared to controls (H_2_O). Next, we activated human platelets with thrombin or CRP and analyzed intracellular Ca^2+^ mobilization in platelets. In the presence of extracellular Ca^2+^, the increase in [Ca^2+^](i) was significantly reduced after stimulation of human platelets with high concentration of thrombin when pre-treated with MK-801 (Figures 5C,D). Reduced Ca^2+^ mobilization was also observed in MK-801 treated human platelets after stimulation with CRP (Figures 5E,F). However, in the absence of extracellular Ca^2+^ (EDTA) we did not detect any alterations in Ca^2+^ mobilization when platelet were activated with thrombin (Figures 5G,H) or CRP (Figures 5I,J) following MK-801 treatment. Taken together, measurements of agonist-induced changes in [Ca^2+^]i revealed a clear reduction of SOCE but not of store release after inhibition of the NMDAR with MK-801 in human platelets.

### 3.7. Reduced ATP release and decreased activation of integrin αIIbβ3 at the surface of human platelets treated with MK-801

**Figure 6:**
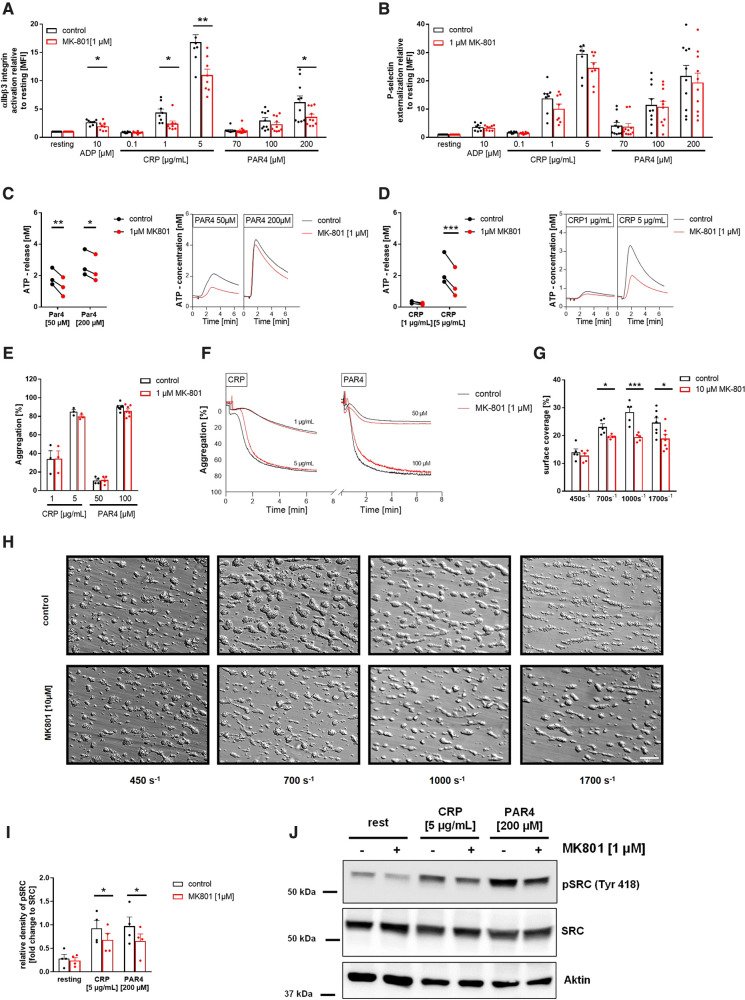
Reduced αIIbβ3 integrin activation, ATP release and thrombus formation of platelets upon inhibition of the NMDAR. (**A**) Activation of αIIbβ3 integrin on the platelet surface with indicated agonists after MK-801 treatment and respective vehicle control (*n* = 10). (**B**) Externalization of P-selectin on the platelet surface with indicated agonists after MK-801 treatment and respective vehicle control (*n* = 10). (**C**) ATP-release measured in platelet releasates after activation with PAR4 activating peptide after MK-801 treatment and respective vehicle control (*n* = 3) with representative measurement. (**D**) ATP-release determined in platelet releasates after activation with CRP and MK-801 treatment and respective vehicle control (*n* = 3). (**E**) Aggregation of washed platelets after stimulation with indicated agonists after MK-801 treatment and respective vehicle control and (**F**) representative aggregation curves (*n* = 3–7). (**G**) Analysis of thrombus formation at indicated shear rates on a collagen matrix with (**H**) representative images after MK-801 treatment and respective vehicle control. (*n* = 5–6). (**I**) Analysis of SRC phosphorylation in isolated human platelets after stimulation with CRP (2 min) and PAR4 (5 min) with (**J**) representative immunoblots (*n* = 4). Depicted are mean values + s.e.m.; *P < 0.05, **P < 0.01 using paired students *t*-test or a 2-way ANOVA with Sidak's multiple comparison test. (CRP, collagen related peptide; Thr,  thrombin; rest, resting state; PAR4, PAR4 activating peptide; ADP, adenosine-diphosphate). Scale bar 50 µm (**H**).

To evaluate the consequences of reduced SOCE on platelet function, we determined platelet activation by flow cytometry using the antibody PAC-1 to detect active integrin αIIbβ3 and using an antibody against P-selectin to determine degranulation of alpha granules. Inhibition of the NMDAR at the membrane of human platelets reduced the number of active integrin αIIbβ3 in response to ADP, CRP and PAR4 peptide at different concentrations (Figure 6A). MK-801 treatment of human platelets did not affect P-selectin exposure of platelets (degranulation of alpha granules) (Figure 6B). In line with the results from knock-out mice, we detected reduced ATP release (dense granule release) upon stimulation with indicated agonists (Figures 6C,D). Because platelet aggregation has been already analyzed in *Grin1^fl/fl^-Pf4-Cre^+^* mice (28), we performed aggregation studies with human platelets and MK-801 treatment. In line with the results from GluN1 deficient knock-out mice, we did not observe any differences when platelet aggregation in response to CRP or PAR4 peptide at different concentrations were analyzed under static conditions (Figures 6E,F). However, under flow, we detected reduced formation of three-dimensional thrombi under all arterial shear rates tested when the NMDAR was blocked by MK-801 (700, 1,000, 1,700 s^−1^) using the well-known flow chamber (Figures 6G,H). In line with results obtained from murine platelets, we analyzed the phosphorylation of Src in human platelets after MK-801 treatment. Inhibition of the NMDAR led to reduced Src phosphorylation following platelet activation with CRP and PAR4 peptide confirming the impact of the NMDAR on Src activation in platelets (Figures 6I,J).

## 4. Discussion

This study reveals that the NMDAR is important for SOCE in platelets but not for store release. Thus, deficiency of GluN1 in mice resulted in reduced Ca^2+^ influx in the presence of extracellular Ca^2+^. Defective Ca^2+^ mobilization in GluN1 deficient platelets affected integrin αIIbβ3 activation, ATP release and Src phosphorylation, but not P-selectin exposure of platelets. These defects were responsible for reduced thrombus formation under flow conditions ex vivo and protection against arterial thrombosis in platelet-specific GluN1 deficient (*Grin1^fl/fl^-Pf4-Cre^+^*) mice.

Platelets express different iGluRs including kainate receptors (KARs), a-amino-3-hydroxy-5-methyl-4-isoxazolepropionic acid receptors (AMPARs), and NMDARs (34, 35). While all of them mediate membrane depolarization by Na^+^ influx, NMDARs yield additional influx of Ca^2+^ upon activation (36, 37). Several studies have provided evidence for glutamate to activate platelets by both AMPAR (38) and KAR activation (39). Treatment of platelets with an AMPAR antagonist as well as platelets from GluR1 knock-out mice do not respond to AMPA. More important, GluR1 deficient mice show prolonged occlusion times after FeCl_3_ induced injury of mesenteric arterioles and impaired hemostasis as reflected by prolonged bleeding times (38). The same phenotype was observed in GluR6-deficient mice suggesting that also the KAR is important for hemostasis and thrombosis (39). Here, we have shown that the NMDARs is likewise important for platelet activation in hemostasis and thrombosis as observed for platelet-expressed KARs or AMPARs in recent years. This suggests that glutamate signaling plays a crucial role in platelet activation and arterial thrombosis. However, glutamate is also present in blood with a concentration in healthy adults ranging from 40 to 60 µM (40). Large amounts of glutamate are also released from activated platelets through exocytosis of dense granules. This increases local plasma glutamate concentrations in platelet-rich plasma to 400–800 µM (38, 41, 42) that might be important for the activation of iGluRs at the platelet membrane. Upon glutamate release from activated platelets, this might also activate other blood cells expressing its receptors, such as red blood cells (43), neutrophils (44), monocytes (45), and lymphocytes (46). It is tempting to speculate that these cells will then contribute to thrombus formation or activate immune responses in thrombo-inflammation.

Already in the late nineties, Franconi and colleagues suggested an anti-aggregatory effect mediated by the NMDAR (47). They found that the synthesis of thromboxane B2 was inhibited by treatment of platelets with NMDA. However, they also found that NMDA increased intracellular Ca^2+^ levels in washed platelets in the presence of 1 mM Ca^2+^. Here, we provided strong evidence that glutamate-induced activation of the NMDAR is sufficient to induce Ca^2+^ mobilization and SOCE because in this study, we only stimulated platelets with standard agonists such as thrombin, PAR4 peptide or collagen/CRP that are known for their ability to induce glutamate release from platelets (41, 42). In contrast, Hearn and colleagues observed Ca^2+^ mobilization only in the presence of NMDA and TG or NMDA and ADP but not with TG or ADP alone (28), although they provided direct evidence for glutamate to be important for Ca^2+^ flux via NMDAR. However, this might be due to the fact that ADP is a weak agonist and glutamate release might be only relevant after platelet stimulation with strong agonists such as collagen or thrombin. Furthermore, Hearn and colleagues found that the NMDAR affects both, Ca^2+^ mobilization via SOCE and via store release. In contrast, we did not detect any alterations in store release in GluN1 deficient platelets, neither with the agonists' thrombin or CRP nor with TG. These results were confirmed by inhibitory studies using human platelets and the NMDAR antagonist MK-801 that reduced Ca^2+^ influx through SOCE whereas store release was unaltered in MK-801-treated platelets. Since the NMDAR is expressed at the plasma membrane, it is tempting to speculate that only Ca^2+^ influx form the extracellular space might be affected by GluN1 deficiency of platelets.

This hypothesis is strengthened by the fact that Orai1, the major SOC channel at the platelet surface, displays major defects in SOCE but not in store release (7)). The defective SOCE translated into reduced αIIbβ3 integrin activation and P-selectin exposure only in response to CRP and CVX but not to G-protein coupled receptor agonists such as thrombin or ADP (7). In contrast, we observed a clear αIIbβ3 integrin defect in platelets with non-functional NMDAR with all agonists tested but no differences in P-selectin exposure suggesting that a defect in Ca^2+^ mobilization in platelets might affect different cellular processes according through which receptor is responsible for Ca^2+^ influx into platelets. Stromal interaction molecule 1 (STIM1) was identified as Ca^2+^ sensor in platelets that is located at the endoplasmic reticulum and relocates to the plasma membrane upon store release. In contrast to Orai1 and NMDAR, STIM1 is important for both, store release and SOCE (7, 48), STIM1 is regulated by Cyclophilin A (CyPA), another important Ca^2+^ regulator in platelets. CyPA deficient platelets displayed defective store release and SOCE (8). Consequently, CyPA deficiency in platelets was responsible for defective integrin activation and degranulation in response to thrombin and ADP but not to CRP or CVX. However, CyPA deficient mice were protected against arterial thrombosis because defective Ca^2+^ mobilization led to reduced thrombus formation as observed for different mice with deficiency in proteins that are known as Ca^2+^ regulators such as Orai1 or STIM1 (7, 48) and NMDAR as shown here.

The regulation of Orai1 by STIM1 has been shown in the past. However, the regulation of the NMDAR might be different and probably not depending on Ca^2+^ release from intracellular stores. To date, the regulation of NMDAR activation is not well understood, although Hearn and colleagues provided direct evidence that glutamate is important for platelet Ca^2+^ influx from the extracellular space because glutamate binding to the GluN2 subunit plays a key role in the activation of the NMDAR besides glycine or D-serine that bind to the GluN1 and GluN3 subunits (28, 49). If STIM1 plays a role in the regulation of the NMDAR induced influx of Ca^2+^ has to be investigated in future to examine if intracellular proteins such as STIM1 are important for NMDAR function or if glutamate/glycine are sufficient for NMDAR activation and NMDAR mediated Ca^2+^ influx into platelets. Furthermore, it is not known to date, if Mg^2+^ ions and membrane depolarization play a role in the regulation of NMDAR activity in platelets. Thus, the regulation of NMDAR activity seems to be very complex and needs further investigation in future.

Here, we provided evidence for NMDAR specific regulation of αIIbβ3 integrin activation, ATP release and SOCE but not platelet degranulation of alpha granules as observed in GluN1 deficient mice. In contrast, Hearn and colleagues detected not only integrin defects but also reduced P-selectin exposure in Grin1 deficient platelets following stimulation with thrombin. This might be due to different platelet protocols or thrombin sources since the authors did not detect any alterations with ADP or CVX. In the present study, we confirmed unaltered P-selectin exposure after thrombin stimulation using PAR4 peptide in different concentrations. Furthermore, the results from murine platelets were confirmed with human platelets where the NMDAR was blocked by MK-801, a classical NMDAR open channel blocker (50). Again, no differences in P-selectin exposure were detected (Figure 6B) thus confirming that the NMDAR does not affect alpha granule release. However, effects of MK-801 can only be observed in activated but not in resting platelets. To date, it is not known if NMDARs are always co-activated upon platelet activation when glutamate is released. Moreover, membrane depolarization is required for current to flow but not sufficient for pore opening (51). Furthermore, we observed integrin defects following CRP stimulation of human platelets upon MK-801 treatment that were not observed with murine GluN1 deficient platelets. Thrombus formation on collagen under flow was not significant at a shear rate of 1,700 s^−1^ using whole blood from GluN1 deficient mice. In contrast, treatment of human platelets with MK-801 led to reduced thrombus formation at all arterial shear rates tested in the flow chamber. These differences between GluN1 deficiency and pharmacological inhibition of NMDAR function in platelets might be due to the fact that MK-801 not only blocks NMDARs, but also nicotinic acetylcholine receptors and serotonin and dopamine transporters that are all expressed in platelets (52, 53). Interestingly, the nicotinic acetylcholine receptors expressed in platelets are known for its ability for Ca^2+^ influx and thus affects platelet activation (53). In contrast to the unknown effects of dopamine uptake by platelets, the role of serotonin in autocrine and paracrine functions of platelets has been extensively investigated in recent years (54). Thus, we cannot exclude that MK-801 exerts its function also on the above mentioned receptors and transporters, respectively.

To date, only data about the signaling mechanism of NMDAR exist in the central nervous system. NMDAR is activated by Src (e.g., via 5HT receptors, mGluRs) (55) that leads to Ca^2±^ influx via NMDAR into target cells. In platelets, Src can be activated by different signaling pathways, e.g., by the major collagen receptor GPVI, thrombin receptor PAR4, GPIb-vWF, fibrinogen-bound integrin αIIbβ3, CLEC-2 etc (56). Thus, it is not surprising that we detected reduced Ca^2±^ mobilization (Figure 1) as well as reduced integrin activation and ATP release (Figure 2) after both, stimulation with CRP and thrombin. The question arises how Src is activated by NMDARs because we detected reduced Src phosphorylation in Grin1 deficient platelets. One can speculate that this is due to a feed-forward loop of NMDAR activation and action because of reduced Ca^2±^ mobilization in Grin1 deficient platelets. Another possible mechanism might be mediated by Focal adhesion kinase (FAK) signaling that has been associated with NMDAR activation in the central nervous system (57). The authors found that FAK signaling plays a role in cerebral ischemia. FAK is close to the NMDARs and also to Src and other proteins. FAK upregulates NMDARs via phosphorylation and increases neuronal damage after cerebral ischemia (57). Thus, it is tempting to speculate that NMDARs are able to activate Src via FAK also in platelets. However, the results presented here clearly show that the loss of NMDAR function affects Src activation because phosphorylation of Src is attenuated after platelet activation (Figures 3A,B).

Furthermore, we detected differences in the phosphorylation of PKC substrates. PKC is a major regulator of platelet granule secretion, integrin activation, aggregation and spreading (58). Following agonist-induced platelet activation, PKC induces the phosphorylation of different substrate proteins to exert downstream effects on platelet secretion, aggregation and exocytosis (59). Here, we detected differences in serine phosphorylation of PKC substrates after PAR4 and CRP (Figure 3) stimulation because the phosphorylation of the PKC substrate with a molecular weight of 75 and 100 kDa was reduced (Figures 3E–J). Thus, CRP and PAR4 peptide induced platelet activation (integrin activation, ATP release) might be not only due to reduced Ca^2±^ mobilization via SOCE but also a result of reduced PKC activity. In parallel with Src activation, PKC might not only be a potential activator of the NMDAR but also be activated by the NMDAR via enhanced Ca2± mobilization in platelets as already suggested by Lutzu and Castillo (55). This again provides strong evidence for a potential feed-forward loop of NMDAR activation and action and a NMDAR-Src-PKC cross-talk in platelets.

Taken together, we identified the NMDAR as important regulator of SOCE that plays a crucial role in Src and PKC substrate phosphorylation, αIIbβ3 integrin activation, the release of ATP and thrombus formation. Protection against arterial thrombosis in *Grin1^fl/fl^-Pf4-Cre^+^* mice suggests that the NMDAR is a potent therapeutic target for the prevention of arterial thrombosis in cardiovascular diseases such as acute myocardial infarction or abdominal aortic aneurysm.

## Data Availability

The original contributions presented in the study are included in the article, further inquiries can be directed to the corresponding author. Requests to access the datasets should be directed to ME; margitta.elvers@med.uni-duesseldorf.de.
