# Supplementary material for: The NMDA receptor regulates integrin activation, ATP release and arterial thrombosis through store-operated Ca2+ entry in platelets
Source: Front Cardiovasc Med. 2023 May 12;10:1171831. doi: 10.3389/fcvm.2023.1171831 (PMC10217778; doi:10.3389/fcvm.2023.1171831)
Supplement: Supplementary file 1 [file Datasheet1.pdf]

Amsterdam UMC
Universitair Medische Centra

Amsterdam UMC, locatie VUmc
Afdeling cardiologie
De Boelelaan 1117
1081 HV Amsterdam

Dr. Christos Bourantas
Specialty Chief Editor
Frontiers in Cardiovascular Medicine: Cardiovascular Imaging

February 14th, 2023

Dear dr. Bourantas,

We are pleased to submit our manuscript entitled "**The role and implications of left atrial fibrosis in surgical mitral valve repair as assessed by CMR: The ALIVE study design and rationale**" for consideration for publication in *Frontiers in Cardiovascular Medicine: Cardiovascular Imaging.*

Patients with mitral valve regurgitation commonly suffer from left atrial remodeling. Development of fibrosis is considered to be an important factor in the atrial remodeling process and is frequently associated with occurrence of arrhythmias. Literature on the presence and extent of left atrial fibrosis in patients suffering from mitral valve regurgitation however, is scarce and its clinical implications remain unknown. Therefore, the ALIVE trial is designed to study left atrial remodeling including fibrosis in patients with severe mitral valve insufficiency prior to and after mitral valve repair surgery.

A better understanding of atrial tissue texture changes (i.e. fibrosis) due to (reverse) remodeling will enhance our pathophysiological understanding of a potentially important arrhthmogenic substrate and may ultimately contribute to patient-specific treatment strategies in patients with mitral valve regurgitation.
In addition, to our knowledge this is one of the first clinical studies to use advanced cardiac MRI 3D LGE techniques beyond the context of primary rhythm disorders.
Therefore, we believe our manuscript is of specific interest to the readership of *Frontiers in Cardiovascular Medicine: Cardiovascular Imaging.*

This manuscript is not under consideration elsewhere and none of the paper's contents have been previously published. All authors have read and approved the manuscript.

On behalf of all authors

Yours sincerely,

Marco J.W. Götte, MD, PhD
